# Incidence of Mild Cognitive Impairment, Conversion to Probable Dementia, and Mortality

**DOI:** 10.1093/geroni/igab046.2445

**Published:** 2021-12-17

**Authors:** Yun Zhang, Ginny Natale, Sean Clouston

**Affiliations:** 1 Renaissance School of Medicine, Stony Brook University, Stony Brook, New York, United States; 2 Stony Brook University, Stony Brook, New York, United States; 3 Renaissance School of Medicine, Stony Brook University, Renaissance School of Medicine, Stony Brook University, New York, United States

## Abstract

Background: Few studies have jointly estimated incidence of MCI, conversion to probable dementia, and mortality. Methods: We used data from six waves of the National Health and Aging Trends Study (2011-2016). Multivariable-adjusted multi-state survival models (MSMs) were used to model incidence upon accounting for misclassification. Results: A total of 6,078 eligible NHATS participants 65 years of age and older were included (average age: 77.49 ±7.79 years; 58.42% females; 68.99% non-Hispanic White). Incidence of MCI was estimated to be 41.0 [35.5, 47.3]/1,000 person-years (PY). Participants converted to probable dementia at a high rate of 241.3 [189.6, 307.0]/1,000 PY, though a small number also reverted from MCI to cognitively normal. Education was associated with lower incidence of MCI and probable dementia, but increased mortality in those with MCI. There were also substantial racial and ethnic disparities in the incidence of MCI and dementia. Conclusions: Our results underscore the relatively common incidence of and conversions between MCI and dementia in community-dwelling older Americans and uncover the beneficial impact of education to withstand cognitive impairment before death.

